# Establishing the Reliability of a Functional Performance Test Battery That Incorporates the QASLS Tool in Pre-Elite Female Field Hockey Players

**DOI:** 10.3390/sports14050198

**Published:** 2026-05-12

**Authors:** Rosalyn Cooke, Lee Herrington, James Martin, Alison Rushton, Nicola Heneghan, Andy Soundy

**Affiliations:** 1 School of Sport, Exercise and Rehabilitation Sciences, College of Life and Environmental Sciences, University of Birmingham, Birmingham B152TT, UK; n.heneghan@bham.ac.uk (N.H.); a.a.soundy@bham.ac.uk (A.S.); 2 The School of Health and Society, Mary Seacole Building, Frederick Road Campus, Broad St, Salford M66PU, UK; l.c.herrington@salford.ac.uk; 3 Institute of Applied Health Research, Public Health Building, College of Medical and Dental Sciences, University of Birmingham, Birmingham B152TT, UK; j.t.martin@bham.ac.uk; 4 School of Physical Therapy, Faculty of Health Sciences, Western University, London, ON N6A 3K7, Canada; arushto3@uwo.ca

**Keywords:** functional performance tests, movement quality, female athletes, field hockey

## Abstract

Pre-elite female field hockey players have a high incidence of lower extremity injury, highlighting the need for practical and reliable screening approaches. A dual assessment combining Functional Performance Tests (FPTs) with movement quality scoring (QASLS) may provide a more comprehensive evaluation; however, its reliability in this population is unclear. Fifteen pre-elite female field hockey players (16.7 ± 0.7 years) completed an FPT battery (anterior reach (AR), single leg drop vertical jump–land (DVJL), single hop for distance (SHFD), side hop (SH)) on two occasions, 28 days apart. Movement quality was assessed by three raters using QASLS. Reliability was evaluated using ICC with 95% confidence intervals (CI), alongside standard error of measurement (SEM), smallest detectable difference (SDD), and percentage exact agreement (PEA). Test–retest reliability varied across tasks (ICC_2,1_ 0.33–0.90), with wide confidence intervals indicating uncertainty in several estimates. AR demonstrated the most consistent reliability, supporting its use for monitoring over time. In contrast, the DVJL and SH showed the greatest variability, likely reflecting higher task complexity, while the SHFD required relatively large performance changes to exceed measurement error. Intra-rater reliability for QASLS was consistent across the FPT battery (ICC_2,k_ 0.79–0.90), whereas inter-rater reliability was more variable (0.38–0.82), indicating rater-dependent differences. PEA demonstrated generally high agreement (60–100%), although lower agreement was observed for pelvic alignment components. These findings support the use of a dual assessment approach as a practicable profiling approach in pre-elite female field hockey, enabling practitioners to identify movement deficits not captured by performance metrics alone. However, variability in complex tasks and between raters highlights the need to consider measurement error and implement standardised rater training when profiling or monitoring performance.

## 1. Introduction

Field hockey players adopt a sport-specific posture characterised by trunk and lower limb flexion, with both upper limbs engaged in stick handling while the ball travels along the ground [1,2]. This posture is maintained during high-speed actions such as running, cutting, and decelerating. Biomechanical analyses have shown that field hockey players demonstrate increased trunk and hip flexion, as well as greater lateral trunk lean during unanticipated sidestepping tasks [3]. These trunk positions influence knee joint loading, contributing to increased knee internal rotation and valgus moments, which are associated with injury risk and may be further exacerbated when upper limb positioning is constrained [4].

Injury surveillance studies in female field hockey, including collegiate cohorts from the 2018/2019 NCAA season, report that lower limb injuries account for approximately 51% of all injuries, with ankle sprains being the most common, alongside concussion [2,5]. Longitudinal data indicate that collegiate players are at a heightened risk of recurrent injury within 61 days of the initial injury, representing a critical window for targeted injury prevention [6]. This is particularly relevant for pre-elite athletes, who represent the developmental pathway to senior international competition. Preventing initial (index) injuries within this population is therefore a priority, as it supports training availability, long-term musculoskeletal health, and performance progression [7,8]. Athlete profiling is a key component of injury prevention strategies, enabling the assessment of physical and movement characteristics through Functional Performance Tests (FPTs) [9].

Functional Performance Tests (FPTs) are structured movement tasks designed to replicate sport-specific demands and are typically assessed using objective performance outcomes such as hop distance, jump height, or completion time [10]. However, poor movement quality and inter-limb asymmetry have also been associated with an increased risk of lower extremity injury (LEI). Traditionally, athlete screening has either considered the performance metrics or the movement quality of the task in isolation. This may account for the lack of success of screening athletes for injury risk using FPTs [9,11].

Despite the multiplanar demands of field hockey and the high burden of LEI, there is limited research examining the use of FPTs within this population [12]. The Y-balance test is one of the few assessments applied in field hockey and has demonstrated associations with LEI risk in community and collegiate players [10,13,14].

A comprehensive evaluation of movement ability should include tasks performed across multiple planes, alongside an assessment of movement quality [15]. FPT typically measure performance in terms of a metric output, and they may fail to identify compensatory or suboptimal movement strategies that occur concurrently, such as lateral trunk lean or knee valgus [16,17]. This may lead to athletes’ performance in the tests being considered sufficient or even good despite the presence of compensatory or suboptimal movement strategies that can also contribute to the athlete’s risk of injury. Evidence from anterior cruciate ligament (ACL) rehabilitation research suggests that reliance solely on performance-based metrics (e.g., distance or height) or limb symmetry indices (LSI) may under- or overestimate an athlete’s readiness to return to sport [15,16,17,18,19]. Additionally, traditional hop test batteries often include multiple horizontal tasks that demonstrate strong inter-test correlations (e.g., single hop and triple hop; ρ = 0.96), potentially limiting their ability to capture diverse movement demands [15,17,18].

Movement quality can be assessed using three-dimensional (3D) motion capture; however, this approach is often impractical in pre-elite environments due to financial and time constraints [12]. Observational tools, such as the Qualitative Assessment of Single-Leg Loading (QASLS), provide a practical alternative and have demonstrated strong validity against 3D motion capture (κ = 0.90 for hop landing; κ = 0.97 for single-leg squat) [20,21,22,23]. The QASLS has been applied to tasks such as the single-leg squat and landing assessments [20,23,24], and adapted versions have been used in hop testing to identify individuals at risk of secondary ACL injury [22]. We define the integration of performance outcomes with observational movement quality assessment as a “dual assessment” approach. The use of this term avoids confusion between descriptors such as quantitative and qualitative or quantity and quality when describing the variables being assessed within a single FPT [18,22,25,26,27]. While dual assessment has been explored in rehabilitation populations, particularly in relation to secondary ACL injury risk, there is limited evidence regarding its application in uninjured populations or within specific sports contexts [15,19,21,25,26,27].

In field hockey, existing research has predominantly focused on elite or community-level athletes [2,3,4,5,6,28,29], with pre-elite female players remaining under-investigated despite their elevated injury risk and biomechanical risk factors for LEI [4,5,6,7]. Previous studies have shown that pre-elite female athletes demonstrate decreased knee flexion and increased knee abduction and moment during dynamic tasks such as landing, which may contribute to their increased injury risk [30,31,32,33].

A dual assessment approach may help to identify both performance deficits and suboptimal movement strategies, thereby informing targeted injury prevention interventions. However, before such an approach can be implemented in practice, it is necessary to establish its reliability within this population.

### Aims

The aim of this study was to evaluate the reliability and practicability of a dual assessment approach that combines Functional Performance Tests with the Qualitative Assessment of Single-Leg Loading (QASLS) in pre-elite female field hockey players. Specifically, this study sought to determine (1) the test–retest reliability of an FPT battery incorporating QASLS, (2) the most reliable performance metric for QASLS application, (3) the intra- and inter-rater reliability of QASLS scoring using still images, and (4) the practicability of implementing dual assessment in a pre-elite team sport environment.

## 2. Materials and Methods

This test–retest, intra- and inter-rater reliability study was designed and is reported in line with the Guidelines for Reporting Reliability and Agreement Studies (GRRAS) [33] to improve the quality of evidence available in relation to measurement properties of this novel application of QASLS with an FPT battery.

### 2.1. Participants

All participants were female field hockey athletes over sixteen years of age and part of the under-eighteen national training squad for a single national governing body (NGB). Participants were not currently injured or completing rehabilitation and were able to complete single-leg hopping and landing activities in multiple planes of motion. Participants were excluded if they had competed at a senior international competition level or regularly trained (>1 training session per month) in the senior training group. Twenty-four pre-elite female field hockey players competing at junior international level were invited and consented to participate in this study.

### 2.2. Sampling and Sample Size

A convenience sampling technique was adopted. A priori power analysis was conducted to determine the reliability of FPT battery when using QASLS. This was calculated using an online sample size calculator [34] based on established methods for reliability studies [34,35]. The expected intraclass correlation coefficient (ICC) was set at 0.70 in line with COnsensus-based Standards for the selection of health Measurement Instruments (COSMIN) guidance on quality of measurement properties [35,36] Alpha level was set at 0.05, with three raters included and a statistical power of 0.80. This indicated that a minimum of 169 observations would be required. Of the twenty-four participants, fifteen participants completed both profiling sessions and were included in the analysis.

The test battery included four FPTs, with twenty expected observations recorded per subject (3 measurements each for left and right limbs in AR, SLDVJ and SHFD (1 measurement for each limb in SH)). This generated 300 observations per session for the fifteen independent participants.

Although this number of observations exceeded the a priori estimate, the number of independent participants (*n* = 15) remains the determinant of between-subject variance, while repeated observations improve precision. In line with contemporary methodological guidance, sample size considerations in reliability studies should account for both the number of participants (n) and the number of repeated measurements or raters (k), as both contribute to the precision of ICC estimates [35]. Therefore, the present study design incorporated multiple trials and raters to optimise precision within a feasible sample size. Given the applied nature of this study within a pre-elite sporting environment, a pragmatic balance between methodological rigour and feasibility was adopted.

### 2.3. Ethical Approval

Participant information and consent were provided and collected utilising the REDCAP data collection system hosted at the University of Birmingham [37]. This study was approved by the University of Birmingham Ethics Committee (ERN_21-1414).

### 2.4. Study Setting

Participants completed the FPT battery over two testing sessions, 28 days apart. Testing sessions took place in an indoor sports hall, and prior to any other hockey training or physical activity. Participants were allocated an identification number, randomly allocated to a test station for one of the FPT, and then proceeded to complete all FPT in a block order. All testing was completed in training footwear and normal hockey training apparel, including shorts/skorts or leggings and a t-shirt. Subjects’ performance in all FPT was filmed using a GoPro Hero 4 camera at 60 fps to enable analysis of movement performance using QASLS.

### 2.5. Data Collection

#### Functional Performance Tests

Four FPTs were selected to be included in the test battery. Selection of the FPTs was based on certainty of evidence for measurement properties, practicability and the requirement to assess movement quantity and quality in frontal and sagittal planes [38,39].

Standardised test protocols were developed based on previously reported methods and aligned to the reporting standards for hop tests [40,41] and are outlined in Table 1. All subjects were allowed to perform a self-directed warm-up that they would normally complete before gym-based training. As this was not a variable of interest, it was not standardised or recorded and may be a source of variation in performance [23].

All subjects completed three practice trials on each leg before anterior reach (AR), single-leg drop vertical jump (DVJL) and single hop for distance (SHFD), and a maximum of 10 s of side hop (SH) on each leg before measured trials. This served as a familiarisation procedure in line with current understanding of the learning effects and score stabilisation for hop tests [40,41]. Subjects self-selected which limb they tested first in each of the FPTs. 

### 2.6. Movement Quality Assessment: Qualitative Analysis of Single-Leg Loading (QASLS)

**Figure 1:**
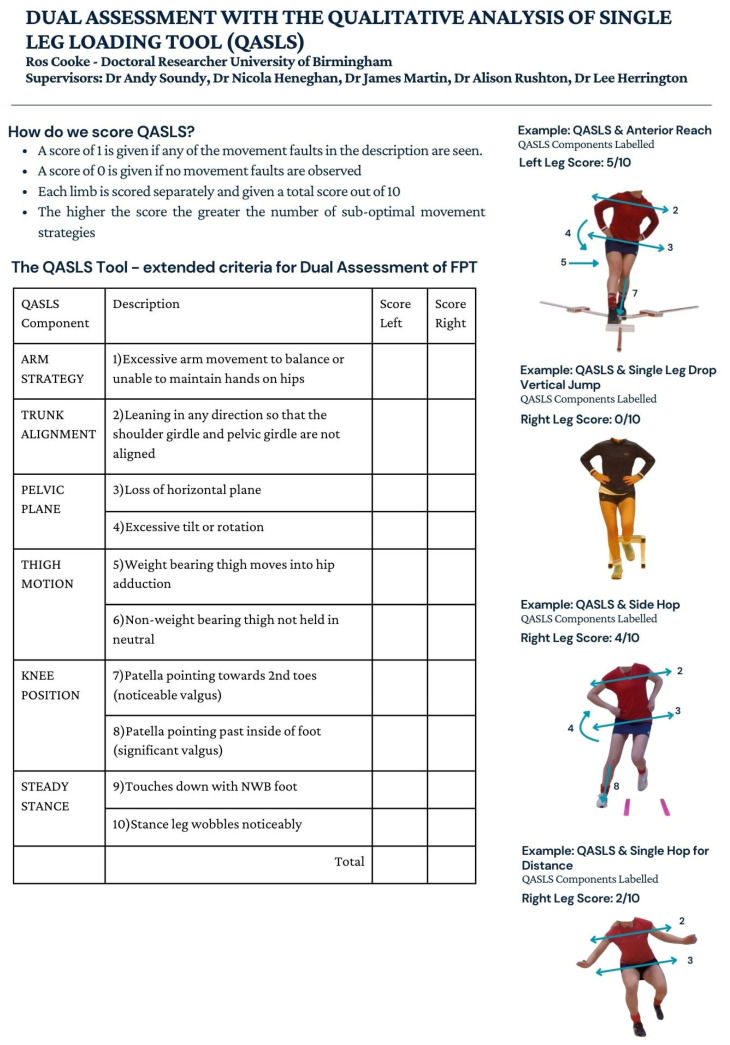
QASLS tool scoring sheet with examples of how to score each FPT. 2.8. Rater characteristics.

Visual assessment of functional movement performance utilising video has been established as a reliable and practicable alternative to 3D motion capture [20,23,24]. The QASLS tool has been developed and validated for assessing the performance of single-leg movement tasks by evaluating the body segment movement strategies. This was initially developed for a single-leg squat [20] but has been utilised in other single-leg activities [24]. The QASLS tool score is calculated by assigning a score of 1 for the presence of suboptimal movement strategies or 0 if absent at each segment as described on the score sheet (Figure 1). The potential range of scores is from 0 to 10, with a higher score indicating greater suboptimal movement in the task.

Within this study, the QASLS tool was applied to a still image instead of video footage. The still images at peak knee flexion were extracted via frame-by-frame analysis by the lead researcher (RC) of the terminal landing or reach for the AR, DVJL and SHFD in line with previously reported methods [16,25,46,47]. For the SH, due to it being a repeated movement task and variation in the number of hops completed by participants, the QASLS was assessed at 5 s intervals throughout the duration of the test, which lasted 30 s. All images were taken from footage captured in the frontal plane only to reflect the practicability of applying observational movement assessment in the field [25].

Additional descriptors were added to the QASLS tool for evaluating the performance of the FPTs where the arms are fixed (AR, DVJ, SH) and in the SH, where the movement demands of the task produce a trunk lean (Figure 1).

Each testing station was allocated a scorer to run the FPT and record the participant’s performance. All scorers were provided with written instructions and verbal guidance on how the FPT was to be conducted and scored for the AR and SHFD. The DVJL and SH scorers oversaw the test but were not required to score each trial. The lead researcher (RC) completed the scoring of the SH and DVJL using video footage and the methods outlined in Table 1. This was conducted after the completion of both testing sessions and QASLS assessments to maintain blinding to performance in the FPT. Information and instructions to scorers were standardised due to the variation in experience and consistency of scorers across the two testing sessions (4 physiotherapists and 2 field hockey technical coaches). All scorers were blinded to the performance of participants outside of the testing station they were involved in and between testing sessions one and two.

Three raters completed the QASLS assessment: the lead researcher (RC, Rater 1) and two additional highly experienced physiotherapists with over 15 years of clinical practice and research experience, including experience with QASLS in athletic populations (Raters 2, 3). Rater training was completed asynchronously with the provision of instructions for QASLS scoring, including modifications to original component descriptions, images to demonstrate these QASLS criteria (Figure 1) and a QASLS scoring sheet within an Excel database. The order of participants when scoring the images was randomised for each FPT to minimise familiarisation. No formal calibration was completed due to all raters’ previous extensive experience with using QASLS. This may have contributed to the observed inter-rater variability and should be considered when interpreting the findings. All raters were blinded to the performance of the participants and completed the scoring independently in a single session.

### 2.7. Data Processing

All measured trials for each FPT from the participants who completed both testing sessions (n = 15) and for each limb (left, right) were utilised to calculate the mean trial (mean of 3 trials), best trial and worst trial. (Supplementary Materials S2 (https://doi.org/10.25500/edata.bham.00001255)). Currently, there is a lack of clear recommendations on which score should be utilised for assessing performance in FPT and therefore all options were included for analysis and to inform which score should be utilised for dual assessment [36].

Intra- and inter-rater reliability of the QASLS tool was calculated based on the performance of 5 randomly selected participants from within the sample by 3 raters. This provided 150 observations per rater, 450 total observations for inter-rater evaluation and 300 observations for intra-rater evaluation. As analysis and scoring take approximately 30 s per observation, this equates to a minimum of 75 min of data processing.

### 2.8. Statistical Analysis

In this study, statistical analyses were performed using STATA 18 (StataCorp 2023). The code used for these analyses is provided in the Supplementary Materials S3 (https://doi.org/10.25500/edata.bham.00001255) to ensure reproducibility and transparency. Test–retest reliability was assessed using intraclass correlation coefficients (ICC_2,1_). This two-way random-effects model with absolute agreement was selected as both participants and testing sessions were considered random factors, and the primary aim was to evaluate absolute agreement between repeated measurements. Assumptions of normality and homoscedasticity were assessed and verified prior to analysis. ICC_2,1_ was calculated for the mean (average of three trials), best trial and worst trial for each limb across sessions one and two for the AR, DVJL, and SHFD. For the SH, the total number of hops (total), number of errors (errors), and performance score (total − errors) were calculated for each limb in both sessions, and ICC_2,1_ was used to determine reliability. Repeated trials in each FPT were not treated as independent observations. ICC values were interpreted as follows: excellent (≥0.90), good (0.75–0.90), moderate (0.50–0.75), and poor (<0.50) [48].

Measurement error was also calculated by reporting the standard error of measurement (SEM = s√(1-R)) and smallest detectable change (SDC = 1.96 × √(2 × SEM)).

Inter-rater reliability of the QASLS composite score was assessed using intra-class coefficients (2,k) [23,24]. A two-way random-effects model with absolute agreement based on the mean of multiple raters. This model was selected as raters were considered representative of a larger population, and the average of their ratings was of interest. ICC was used as the data were considered to be continuous.

To investigate agreement between raters across each component of the QASLS, percentage exact agreement (PEA) was calculated using the formula PEA = (A/N) × 100P, where A represents the number of agreements between the raters, and N is the total number of ratings. PEA quantifies the proportion of identical ratings between or within raters for each component of the QASLS tool, which provides insight into how consistently raters interpret and score movement performance. Interpretation of what is an acceptable PEA% is difficult due to a lack of interpretation guidance, but it has been previously used when evaluating rater agreement for components of QASLS. (23) In line with previous analysis of intra- and inter-rater reliability of the QASLS tool, the following interpretations of PEA were applied: 50–60%: moderate; 61–75%: substantial; and 76–100%: near perfect [23,24]. Intra-rater reliability of the QASLS composite score was analysed using intra-class coefficients (ICC 2,k), and PEA was calculated for the individual components of the QASLS score and interpreted as previously described [23,24].

## 3. Results

Test–retest reliability included fifteen subjects (age: 16.7 ± 0.73; height: 167.9 cm ± 8.08; weight: 61.6 kg ± 7.26) across two sessions, twenty-eight days apart. A total of 300 observations were used to calculate the mean, best and worst trials for each limb for each participant. A total of 450 observations (participants, n = 5; raters, n = 3; observations per participant, n = 30) were used to assess inter-rater reliability, and 300 for intra-rater reliability analysis of the QASLS tool. Supplementary Materials S2 (https://doi.org/10.25500/edata.bham.00001255) contains the raw data of participants’ performance in the FPT and QASLS.

### 3.1. Test–Retest Reliability of FPT Battery

Descriptive statistics and test–retest reliability indices for all FPT are presented in Table 2. Measurement error is reported in Table 3, which presents SEM and SDD across trial comparisons, limbs, and testing sessions, providing additional insight into the consistency of repeated measures.

*Anterior Reach (AR)*

AR demonstrated ICC_2,1_values that ranged from 0.67 to 0.88 across all measures and limbs. Mean reach distance point estimates were relatively high (left: ICC_2,1_ 0.85; 95% CI, 0.54–0.95; right ICC_2,1_ 0.88; 95% CI, 0.64–0.96). However, the confidence intervals are moderately wide, indicating some uncertainty in these estimates. The best and worst trials achieved ICC_2,1_values ranging from 0.71 to 0.73 and 0.67 to 0.70, respectively. The confidence intervals were wider than the mean trial, indicating greater imprecision, despite the point estimates being classed as moderate.

SEM between sessions was 2.15–2.36 cm (left) (SDC 8.43–9.24 cm) and 2.12–3.66 cm right (SDC 8.31–14.34 cm). Despite the small SEM, the SDC scores indicate that a change in score of at least 8.31 cm is required to reflect a meaningful change. These findings suggest that the AR could be suitable for monitoring performance changes over time.

*Drop Vertical Jump–Land (DVJL)*

Jump height demonstrated differences between limbs. For the left limb, ICC values ranged from 0.79 to 0.90; however, the associated CIs were wide for mean, best and worst scores (ICC_2,1_ mean: 0.90, 95% CI: 0.71–0.97; best: 0.79, 95% CI: 0.48–0.92; worst: 0.80, 95% CI: 0.50–0.93). This indicates some uncertainty in the point estimates. The right limb demonstrated lower ICC values with wide confidence intervals, which included negative values (ICC_2,1_ mean: 0.65, 95% CI: −0.10–0.88; best: 0.39, 95% CI: −0.15–0.74; worst: 0.48, 95% CI: −0.05–0.79). This indicates imprecision in these reliability estimates.

SEM between sessions was similar between limbs, ranging from 1.43 to 1.76 cm for the left (SDC 5.61–6.90 cm) and 1.51–1.54 cm for the right (SDC 5.90–6.05 cm).

These values indicate that changes greater than approximately 6 cm are required to reflect true change beyond measurement error. Differences between limbs may reflect the complexity of this movement task, with greater variability in movement strategy between testing sessions, and not measurement error alone.

*Single Hop for Distance (SHFD)*

SHFD demonstrated ICC_2,1_ values ranging from 0.52 to 0.81 across measures and limbs. Despite these point estimates, the associated confidence intervals were wide, indicating uncertainty (ICC_2,1_ mean: 0.73–0.81, 95% CI: 0.16–0.95; best: 0.65–0.72, 95% CI: 0.21–0.90; worst: 0.52–0.67, 95% CI: 0.01–0.88). SEM between sessions was similar between limbs, ranging from 5.64 to 8.93 cm for the left and 5.06–9.23 cm for the right. SDC values (left: 22.11–35.00 cm; right: 19.85–36.17 cm) indicate that relatively large changes (approximately 20–36 cm) are required to reflect true change beyond measurement error. These findings suggest that SHFD may not be the most suitable FPT when monitoring uninjured populations, as only large changes in hop distance can be interpreted with confidence.

*Side Hop (SH)*

The SH demonstrated ICC_2,1_ values ranging from 0.33 to 0.64 across all outcomes and both limbs. For total hops, ICC_2,1_ values were 0.63–0.64, with wide confidence intervals for both sides (ICC_2,1_ left: 0.64, 95% CI: 0.19–0.86; right: 0.63, 95% CI: 0.21–0.86). The number of errors and score point estimates were both lower with wide confidence intervals that included negative values (errors: ICC_2,1_ left: 0.40, 95% CI: −0.06–0.74; right: 0.33, 95% CI: −0.12–0.71; score: ICC_2,1_ left: 0.53, 95% CI: 0.07–0.81; right: 0.43, 95% CI: −0.04–0.75).

SEM between sessions was similar between limbs, with values of 3.72 for the left and 4.16 for the right (SDC left: 10.32; right: 11.52). These values indicate that changes greater than 10–12 hops are required to reflect true change in performance beyond measurement error. The relatively wide confidence intervals and higher measurement error observed in the side hop may partly reflect greater movement variability in the pre-elite population and the dual task demands of this FPT, which likely amplifies this variability, contributing to less consistent performance.

### 3.2. Intra-Rater Reliability of QASLS

Intra-rater reliability of the composite score of the QASLS tool was assessed for each of the FPTs in the test battery. ICC_2,1_ values ranged from 0.79 to 0.90, with relatively narrow confidence intervals for AR (ICC_2,1_ 0.90; 95% CI: 0.81–0.95) and DVJL (ICC_2,1_ 0.90; 95% CI: 0.79–0.95), indicating greater precision in these estimates. For SH (ICC_2,1_ 0.79; 95% CI: 0.66–0.87) and SHFD (ICC_2,1_ 0.83; 95% CI: 0.62–0.92), ICC values were slightly lower, with wider confidence intervals, suggesting comparatively greater uncertainty. PEA for the individual components of the QASLS ranged from 83.3% to 100% for AR, DVJL, and SHFD and from 66.7% to 100% for SH, indicating generally high levels of agreement across components. Detailed PEA results are presented in Supplementary Materials S4. (https://doi.org/10.25500/edata.bham.00001255).

### 3.3. Inter-Rater Reliability of QASLS

Table 4 presents the inter-rater reliability of QASLS composite scores across the FPT battery. ICC_2,k_ estimates varied by test and rater combination. For AR, comparisons involving Rater 2 appeared to show greater divergence compared to Raters 1 and 3, with ICC values ranging from 0.38 to 0.82 and wide confidence intervals (95% CI: −0.23 to 0.91), indicating substantial imprecision. A similar pattern was observed for SHFD, where ICC values for Raters 1 and 2 (0.48; 95% CI: −0.23–0.80) and Raters 2 and 3 (0.46; 95% CI: −0.14–0.81) were accompanied by wide confidence intervals that included negative values. In contrast, agreement between Raters 1 and 3 was higher (ICC_2,k_ 0.77; 95% CI: 0.52–0.89), although the confidence interval remained moderately wide. For DVJL (ICC_2,k_ 0.70–0.85) and SH (ICC_2,k_ 0.54–0.78), ICC values were higher overall; however, confidence intervals remained wide and, in some cases, included negative values, indicating continued uncertainty in these estimates. Overall, inter-rater reliability estimates should be interpreted with caution due to the small subsample (n = 5) and the width of the confidence intervals, which reflect substantial uncertainty in the precision of these estimates.

Inter-rater agreement of individual QASLS components (Supplementary Materials S4 (https://doi.org/10.25500/edata.bham.00001255)) demonstrated moderate to perfect agreement across all FPTs (PEA: 60.0–100%). Arm strategy (Component 1) in SHFD showed substantial agreement (PEA: 70.0–73.3). Lower agreement was observed for Component 4 (pelvic tilt/rotation), particularly in AR, SH, and SHFD (PEA 60.0–78.3), while SH demonstrated additional components with lower agreement, including loss of horizontal pelvic plane (Component 3, PEA: 66.7%) and position of the non-weight-bearing thigh (Component 6, PEA: 68.3%).

## 4. Discussion

This study aimed to evaluate the test–retest, intra-rater, and inter-rater reliability of an FPT battery incorporating the QASLS tool within a dual assessment approach in a cohort of pre-elite female field hockey players. To the authors’ knowledge, this is the first study to investigate this combined quantitative and qualitative assessment approach in an uninjured, high-risk athletic population. The methodology is novel in applying expanded QASLS criteria to still frontal plane images across multiple functional tasks performed in both sagittal and frontal planes. Establishing the measurement properties of this approach is therefore essential to support its use in clinical and performance settings [20,23,39,40].

### 4.1. Which Score to Use in FPT?

**Figure 2:**
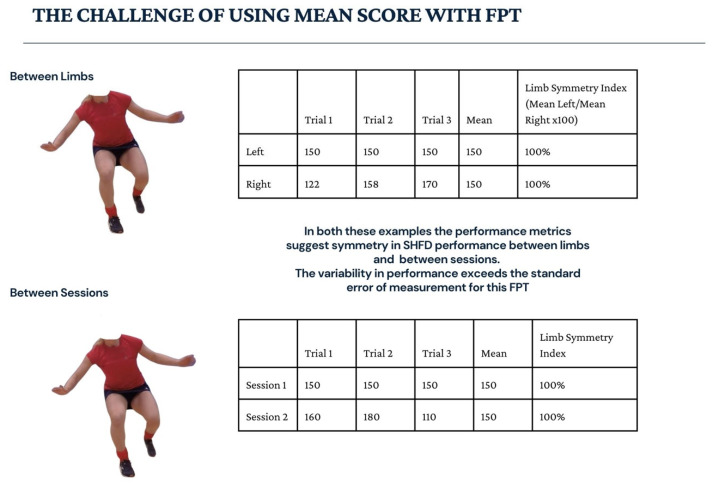
The challenge of using mean score with FPTs.

Mean scores of performance metrics from FPTs are commonly used to assess performance and calculate the limb symmetry index (LSI) [40]. These methods improve reliability, as the measurement error is reduced, but may lead to an overestimation of an individual’s ability and produce misleading results [26,49,50]. As illustrated in Figure 2, equivalent mean scores can mask substantial variability between trials, limbs or sessions, potentially resulting in misleading interpretations of symmetry and performance.

This is particularly relevant given the variability in performance metrics in the FPT battery observed in this study across trials and sessions. Reliance on mean values alone may obscure inconsistencies in performance and reduce sensitivity to detect meaningful deficits or changes in performance. This reinforces the importance for practitioners to consider reliability assessments and what this tells us about the variability (ICC/95% CI) and the precision (SEM/SDC) of the FPTs when using them for profiling or monitoring purposes.

Temporality of performance in FPTs for uninjured populations is not well reported within the literature, with a need for longitudinal studies with repeated measures using FPT with known measurement properties [9,10,15,39,40].

In line with recommendations to improve reporting standards of hop tests [39,40], this study examined best and worst trial performance alongside mean values. Best and worst scores for both AR and SHFD demonstrated ICC_2,1_ values ranging from 0.67 to 0.73 and 0.52 to 0.72, with wider confidence intervals than mean scores, indicating imprecision. The mean score ICC for both these tests were consistent with previously reported studies, which found excellent test–retest reliability (AR ICC_2,1_ 0.84–0.93; SHFD ICC_2,1_ 0.92–0.97) [49,51]. The differences in ICC for best and worst scores may arise due to previous studies using mean scores and the cohorts being mixed-sex, elite and collegiate-level athletes who demonstrate more consistent movement performance [30,31,32]. The lower reliability values reported in this study may suggest that best and worst trials may be more sensitive to movement variability, particularly in pre-elite populations. Greater movement variability and asymmetry have been observed in closed skill tasks such as hop tests in female pre-elite populations [50,52,53]. These asymmetries appear to reduce with increasing chronological age, movement skill and strength [52,53,54].

For DVJ, between-limb difference was observed in the ICC values, with the left limb achieving higher ICC values across mean, best and worst trials compared to the right. Wide confidence intervals were reported for both limbs, with negative values observed in the right limb. The between-limb difference is only observed in this FPT and may be accounted for by the asymmetrical movement demands of field hockey [1,2,3,4,5,6] and the movement variability of pre-elite female athletes [30,31,50,52,53,54].

In contrast to the other FPTs, the SH is a continuous task and does not allow for best and worst trial selection. All metrics assessed found lower ICC values, with wide confidence intervals and SEM and SDC scores requiring a large change (10–12 hops). This likely reflects the increased complexity of the movement task and the associated increase in variability in its execution within and between sessions in a pre-elite female population with developing neuromuscular control [45,52,53,54]. These results report lower ICC_2,1_values than previously found with a collegiate cohort (n = 14), where test–retest reliability was good to excellent (ICC_2,1_ left = 0.96, right = 0.84) [51]. The differences in our results could be in part due to the age and sex differences in the cohorts and the time interval between testing sessions (one week versus one month). As athlete profiling is not routinely completed on a weekly basis, it is important to indicate the stability of measurement over longer time periods, which are representative of the sports environment.

Collectively, these findings suggest that in uninjured pre-elite female populations, the reliance on mean score in a single FPT, although demonstrating greater reliability, may be insufficient when assessing or monitoring performance over time. Small performance improvements may not be differentiated from measurement error in the SHFD, DVJ and SH, which may reflect the movement task complexity and challenge for this population. A dual assessment approach that incorporates both performance variability (best/worst trial) and movement quality (QASLS) may provide a more comprehensive evaluation of an athlete’s function, which is considered crucial to injury risk assessment and rehabilitation monitoring [7,15,17,20,21,22,23,24,25,26].

### 4.2. Measurement Error and Clinical Interpretation

Measurement error has important implications when utilising an FPT, as it informs the practitioner if they have observed a true change in performance. These measurement properties are not commonly poorly reported [35,46,49]. SEM and SDD values varied considerably across the FPT, with DVJL demonstrating the lowest SEM and SHFD the highest. The relatively small SDD values observed for DVJL (5–8 cm) indicate that relatively small changes in performance can be interpreted as true change.

SEM is not readily reported in the literature, despite multiple validity and reliability reports for the My Jump app [55,56].

In contrast, SHFD demonstrated substantially larger SDC values (up to ~53 cm in Session 1 and ~20–36 cm in Session 2), indicating that only large changes in performance exceed measurement error. This limits the sensitivity of this test for detecting smaller, yet potentially meaningful, changes, particularly in uninjured populations.

The reduction in measurement error observed between sessions for SHFD may reflect a learning or familiarisation effect, whereby participants demonstrated greater consistency following repeated exposure. This aligns with the previous literature reporting stabilisation of performance after multiple trials [10,15,40,41]. However, despite this improvement, measurement error remained high relative to other tests.

SH demonstrated moderate measurement error (SEM 3.72–4.16 hops), with SDC values indicating that changes of approximately 10–12 hops are required to represent true change. This aligns with previous reported research within collegiate and pre-elite Scandinavian populations (SEM 0.5–5.4 hops) [45,51]. These findings emphasise the importance of considering measurement error when interpreting changes in performance so that normal variability is not interpreted as true change.

### 4.3. Is QASLS a Reliable Tool?

Intra-rater reliability of the QASLS composite score produced ICC_2,k_ values from 0.79 to 0.90 across the FPT battery. Confidence intervals were narrower for AR and DVJL compared to SH and SHFD, indicating greater precision for these tasks. These findings suggest that the QASLS tool can be applied consistently by the same assessor, although variability may increase with task complexity.

Inter-rater reliability varied across tests and rater combinations. ICC_2,k_ values ranged from 0.38 to 0.82 for AR and 0.46 to 0.77 for SHFD, with wide confidence intervals that frequently included negative values, indicating substantial uncertainty in these estimates. For DVJL and SH, ICC values were higher overall (DVJL ICC_2,k_ 0.70–0.85; SH ICC_2,k_ 0.54–0.75) across all rater combinations; however, confidence intervals remained wide, again reflecting imprecision. These findings should be considered exploratory due to the small subsample (n = 5) and the instability of the ICC estimates. AR is comparable to a single-leg squat task, and previously, studies have found poor inter-rater reliability when applying QASLS to this task [23,24]. Rater dependence variability was seen in the results of this study, with Rater 2 consistently demonstrating greater divergence in scoring compared to Raters 1 and 3. This suggests that structured rater training and calibration between raters may be beneficial to improve the consistency of application of QASLS in different FPT.

Considering the components of the QASLS score through PEA enables us to understand the level of agreement between raters and provide context to ICC measurements. An example of this is anterior reach, where PEA for QASLS component four (pelvic plane excessive tilt or rotation), where all rater combinations only achieved substantial agreement (%PEA 63.3–76.7), and this lack of agreement would support the observed relatively poorer ICC scores.

PEA is an indication of measurement error when the data are considered to be ordinal or nominal and is therefore the most appropriate for use with the QASLS score, where the components are considered to be ordinal [23]. Lower agreement was consistently observed for pelvic plane assessment (Component 4), particularly in AR, SHFD and SH.

Previous studies have not found this component to be a source of rater disagreement [23,24]. Analysis of the pelvis using gold standard 3D motion capture methods has also found the greatest variation when modelling pelvic motion within the transverse plane; it is therefore unsurprising that this is a source of variability in observational methods [11,19]. Arm positioning (QASLS component one) during the single hop for distance lacked agreement amongst the raters (%PEA 70–73.3) and may result from the ambiguity of the descriptor ‘excessive’, which is open to subjective interpretation.

It is the only FPT in the battery where arm position is not fixed, and a variety of positions have been observed. In line with the findings from Parry [23], agreement between raters about non-weight-bearing thigh position during the SH was lower (%PEA 68.3). Due to trunk position and frontal plane demands of this task, the position of the non-weight-bearing thigh varies as subjects try to stabilise through adducting this limb, which is sub-optimal [20,21,22,23,24]. Component 10 (steady stance) demonstrated perfect agreement across all tests, likely reflecting its limited applicability when using still images and suggesting redundancy within the current methodology.

Overall, these findings suggest that inter-rater reliability findings should be considered as exploratory, given the small subsample (n = 5) and wide confidence intervals associated with the different rater combinations. While QASLS demonstrated good intra-rater reliability, inter-rater reliability is more variable and influenced by both task characteristics and component-specific interpretation. Enhancing descriptor clarity and implementing structured rating training and calibration may improve consistency.

### 4.4. Strengths and Limitations of This Study

This study provides novel insight into the reliability of a dual assessment approach combining performance metrics and movement quality assessment within a field-based FPT battery. To enhance the robustness of the methods and analysis, this study was developed and reported using the GRRAS guidance [33]. The use of multiple raters, repeated testing sessions, and a sport-specific cohort enhances ecological validity and supports the applicability of findings to real-world sports settings. Furthermore, this study was conducted using accessible equipment and methods, enhancing practicability and supporting clinical translation. The reliability results presented here are sufficient according to COSMIN criteria for good measurement properties to support the use of these methods to enable dual assessment of FPT [36].

However, several limitations should be considered. This study included a relatively small sample size (n = 15), which may limit the precision and stability of reliability estimates despite the number of observations collected. Repeated trials were aggregated for analysis, and this may still introduce some dependency in the data structure. Although confidence intervals have been reported to reflect this uncertainty, future studies with larger cohorts are required to confirm these findings.

Inter-rater reliability was assessed using a subsample of five participants, which may reduce the generalisability of these results and should be interpreted with caution. Additionally, while steps were taken to ensure appropriate statistical handling, the use of repeated trials within participants may introduce some dependency in the data structure, which could influence reliability estimates. Participants were all pre-elite female field hockey players, and findings may not be directly transferable to other populations.

The use of still frontal plane images only for QASLS assessment may have influenced reliability outcomes. Scoring of components that assess movement in the sagittal and transverse planes may have been compromised with the use of a single frontal plane camera angle. Multicamera analysis would be a solution, although it may not always be practicable in field-based settings due to the time and resource requirements.

Pelvic motion accounted for the greatest disagreement between raters during AR, SHFD and SH. A marker on the anterior superior iliac spine and pelvic crest may improve visualisation of the pelvis and agreement between raters.

The rater training used in this study and the absence of calibration between raters may also have contributed to variability in scoring, particularly given the raters’ prior experience with the tool. Rater populations also need to be considered, and within this study, only physiotherapists were involved. Although physiotherapists are trained to evaluate, assess and quantify movement through their undergraduate training, this is often concerning normal movement tasks and not sporting actions. It may therefore be wrong to assume that physiotherapists are the only profession within the sports science and medicine team that can deliver analysis in this area.

While the applied setting enhances ecological validity, it inherently reduces experimental control, which likely contributed to variability in both performance and scoring outcomes. In addition, the absence of standardised warm-up procedures, although intended to reflect real-world practice, represents a further source of uncontrolled variability. Although participants were familiar with the tasks and completed standardised familiarisation trials, differences in individual preparation cannot be ruled out as a contributing factor to performance inconsistency. Future research should prioritise greater standardisation of FPT protocols, alongside investigation of learning effects and the implementation of structured rater training, to strengthen the assessment of FPT reliability.

## 5. Conclusions

This study demonstrates that a dual assessment approach combining Functional Performance Tests (AR, DVJL, SHFD, SH) with an observational movement quality tool (QASLS) applied to still images is feasible and demonstrates variable but acceptable reliability in line with COSMIN guidance. Reliability outcomes varied across tests and raters, with intra-rater reliability generally higher than inter-rater reliability, highlighting the influence of rater consistency. Given the small sample size, particularly for inter-rater analysis, further research in larger cohorts is required to confirm these findings. The findings also indicate that pre-elite female athletes exhibit variability in movement performance across functional tasks, which is reflected in the SEM and SDD values reported. Consequently, only changes exceeding the SDD should be interpreted as true performance changes rather than normal measurement variability. Observational movement assessment tools, such as QASLS, provide valuable insight into the strategies athletes use to perform movement tasks and may help inform targeted injury prevention and rehabilitation interventions. However, when used across multiple raters, standardisation and structured training are required to improve consistency and ensure accurate interpretation in both clinical and performance settings.

## Figures and Tables

**Table 1:** Functional Performance Test protocols.

Test	Equipment	Testing Process and Scoring	Indication for Repeat Test
**Anterior Reach (AR)** **[13,42]**	Tape measure Tape to mark floor 1× camera 1× tripod Y-balance testing lit	3 practice trials each side with minimum of 1 min rest Subject selects leg to test first Completes 3 measured trials on first leg and then repeats on other side Distance pushed measured to nearest 0.5 cm Scoring completed at time of testing	Kicking push box Not returning to starting position under control Touching down during reach Foot on top of stance plate
**Single-Leg Drop Vertical Jump (DVJL)** **[16,43]**	Tape measure Tape to mark floor 1 × camera 1 × tripod 30 cm box Landing area marked 30 cm in front of box with a strip of tape	3 practice trials each side with minimum of 1 min rest Subject selects leg to test first Completes 3 measured trials on first leg and then repeats on other side Landing to be held for at least 2 s on completion of DVJ Scoring completed post testing using video footage and My Jump Lab app to calculate: Jump height Contact time Flight time Reactive strength index (RSI)	Loss of balance–steps out of landing Extra hop on landing Touching down with either contralateral leg or with hand
**Single Hop for Distance (SHFD)** **[19]**	Tape measure Tape to mark floor 1 × camera 1 × tripod Tape measure set to 250 cm and secured to the floor with tape	3 practice trials each side with minimum of 1 min rest Subject selects leg to test first Completes 3 measured trials on first leg and then repeats on other side Landing to be held for at least 2 s Measurement taken from heel of landing foot Distance measured to the nearest cm Scoring completed at time of testing	Loss of balance–steps out of landing Extra hop on landing Touching down with either contralateral leg or with hand
**Side Hop (SH)** **[44,45]**	Tape measure Tape to mark floor 2 lines of 1 m 40 cm apart 1 × camera 1 × tripod Timer	Up to 10 s practice each side with minimum of 1 min rest before completing testing Completes 1 set on each leg Self-selected rest between each side minimum of 1 min Subject selects leg to test first Scoring completed post testing using video footage to calculate: Total hops; Total errors; Adjusted score (total hops–total errors); % error (total errors/total hops × 100)	Loss of balance during the test Forgets to keep hands on hips

**Table 2:** Descriptive statistics (mean ± SD) and reliability metrics (ICC, 95% CI, classification) for Functional Performance Test battery.

**Functional Performance Test**	**Left**											
	**Mean of 3 Trials (SD)**	**Mean ICC**	**Mean CI 95%**	**Class**	**Best Trial**	**Best ICC**	**Best CI 95%**	**Class**	**Worst Trial**	**Worst ICC**	**Worst CI 95%**	**Class**
**AR**	61.9 (±5.6)	0.85	(0.54–0.95)	Mod–Exc	63.9 (±5.6)	0.73	(0.35–0.90)	Poor–Exc	59.9 (±5.9)	0.67	(0.26–0.88)	Poor–Good
**(distance cm)**												
**DVJL**	13.8 (±2.5)	0.90	(0.71–0.97)	Good–Exc	15.3 (±2.7)	0.79	(0.48–0.92)	Poor–Exc	12.5 (±2.5)	0.80	(0.50–0.93)	Mod–Exc
**(height cm)**												
**SHFD**	157.5 (±15.5)	0.73	(0.16–0.91)	Poor–Exc	162.5 (±15.6)	0.65	(0.21–0.87)	Poor–Good	152.4 (±15.58)	0.52	(0.01–0.81)	Poor–Good
**(distance cm)**												
	**Total (SD)**	**Total ICC**	**Total CI 95%**	**Class**	**Errors (SD)**	**Errors ICC**	**Errors CI 95%**	**Class**	**Score (SD)**	**Score ICC**	**Score CI 95%**	**Class**
**SH (count)**	56.0 (±5.9)	0.64	(0.19–0.86)	Poor–Good	9.1 (±4.7)	0.4	(−0.06–0.74)	Poor–Mod	46.9 (±8.02)	0.53	(0.07–0.81)	Poor–Good
**Functional Performance Test**	**Right**											
	**Mean of 3 Trials (SD)**	**Mean ICC**	**Mean CI 95%**	**Class**	**Best Trial**	**Best ICC**	**Best CI 95%**	**Class**	**Worst Trial**	**Worst ICC**	**Worst CI 95%**	**Class**
**AR**	61.7 (±6.1)	0.88	(0.64–0.96)	Mod–Exc	64.1 (±6.2)	0.71	(0.35–0.89)	Poor–Good	59.4 (±6.7)	0.70	(0.29–0.89)	Poor–Good
**(distance cm)**												
**DVJL**	13.67 (±2.49)	0.65	(−0.10–0.88)	Poor–Good	15.01 (±2.80)	0.39	(−0.15–0.74)	Poor–Mod	12.46 (±2.47)	0.48	(−0.05–0.79)	Poor–Good
**(height cm)**												
**SHFD**	156.96 (±13.45)	0.85	(0.54–0.95)	Mod–Exc	163.03 (±14.20)	0.72	(0.36–0.90)	Poor–Exc	150.83 (±14.40)	0.67	(0.26–0.88)	Poor–Good
**(distance cm)**												
	**Total (SD)**	**Total ICC**	**Total CI 95%**	**Class**	**Errors (SD)**	**Errors ICC**	**Errors CI 95%**	**Class**	**Score (SD)**	**Score ICC**	**Score CI 95%**	**Class**
**SH (count)**	54.2 (±6.77)	0.63	(0.21–0.86)	Poor–Good	9.43 (±4.85)	0.33	(−0.12–0.71)	Poor–Mod	44.777 (±7.78)	0.43	(−0.04–0.75)	Poor–Good

FPT: Functional Performance Test. AR: anterior reach. DVJL: single-leg drop vertical jump–land. SHFD: single hop for distance. SH: side hop. Mean: mean of 3 trials. Best: best scoring trial. Worst: worst scoring trial. Total: total number of hops. Errors: number of errors. Score: score = total number of hops–errors. ICC: intraclass correlation coefficients (ICC^2,1^). CI 95%: 95% confidence interval for ICC. Class: classification of ICC. Exc: excellent ≥ 0.90. Good: good 0.75–0.90. Mod: moderate 0.50–0.75. Poor: poor < 0.50.

**Table 3:** Standard error of measurement (SEM) and smallest detectable difference (SDD) across trials, limbs, and testing sessions for Functional Performance Tests

		Testing Session 1				Testing Session 2			
		Left		Right		Left		Right	
	Trial	SEM	SDC	SEM	SDC	SEM	SDC	SEM	SDC
Anterior Reach	1v2	2.75	10.77	1.87	7.32	2.13	8.34	3.46	13.57
	1v3	1.89	7.41	2.15	8.41	2.07	8.11	4.28	16.78
	2v3	2.44	9.55	2.35	9.22	2.26	8.85	3.23	12.66
	Mean	2.36	9.24	2.12	8.31	2.15	8.43	3.66	14.34
Drop Vertical Jump–Land	1v2	2.16	8.47	1.64	6.43	1.43	5.62	1.28	5.01
	1v3	1.38	5.42	1.61	6.29	1.38	5.40	1.43	5.59
	2v3	1.74	6.81	1.27	4.99	1.48	5.79	1.92	7.53
	Mean	1.76	6.90	1.51	5.90	1.43	5.61	1.54	6.05
Single Hop for Distance	1v2	13.49	52.87	11.19	43.87	4.82	18.88	4.18	16.39
	1v3	6.76	26.50	9.36	36.70	7.11	27.85	5.13	20.10
	2v3	6.54	25.63	7.13	27.94	5.00	19.59	5.89	23.07
	Mean	8.93	35.00	9.23	36.17	5.64	22.11	5.06	19.85
Side Hop		3.72	10.32	4.16	11.52				

**Table 4:** Inter-rater reliability of QASLS composite score by FPT.

QASLS Composite Score	Rater 1v2	Classification	Rater 1v3	Classification	Rater 2v3	Classification
**Anterior Reach**	0.38 (−0.23–0.71)	Poor–Moderate	0.82 (0.61–0.91)	Moderate–Excellent	0.38 (−0.23–0.71)	Poor–Moderate
**Drop Vertical** **Jump–Land**	0.85 (0.53–0.93)	Moderate–Excellent	0.76 (0.48–0.88)	Poor–Good	0.70 (−0.16–0.90)	Poor–Good
**Side Hop**	0.75 (0.31–0.88)	Poor–Good	0.78 (0.54–0.89)	Moderate–Good	0.54 (−0.15–0.79)	Poor–Good
**Single Hop** **for Distance**	0.48 (−0.23–0.80)	Poor–Good	0.77 (0.52–0.89)	Moderate–Good	0.46 (−0.14–0.81)	Poor–Good

## Data Availability

All data generated or analysed for this study are included in this published article and within the Supplementary Materials, which are linked to the UBIRA eData Repository hosted by the University of Birmingham (https://doi.org/10.25500/edata.bham.00001255).

## Supplementary Materials

The following supporting information can be downloaded at: https://www.mdpi.com/article/10.3390/sports14050198/s1 or https://doi.org/10.25500/edata.bham.00001255. S1: Functional performance testing procedures. S2: Raw data of participants’ performance in FPT and QASLS scores. S3: Stata syntax for statistical analysis. S4: Intra- and inter-rater reliability of individual QASLS components.

## Abbreviations

AR	anterior reach direction of the Y-balance test
COSMIN	COnsensus-based Standards for the selection of health Measurement INstruments
DVJL	drop vertical jump–land test
FPT	Functional Performance Test
GRRAS	Guidelines for Reporting Reliability and Agreement Studies
ICC	intraclass correlation coefficients
NGB	national governing body
PEA	percentage exact agreement
QASLS	Qualitative Assessment Single-Leg Loading
SH	Side Hop Test
SHFD	Single Hop for Distance Test
