# Supplementary material for: Establishing the Reliability of a Functional Performance Test Battery That Incorporates the QASLS Tool in Pre-Elite Female Field Hockey Players
Source: Sports (Basel). 2026 May 12;14(5):198. doi: 10.3390/sports14050198 (PMC13210639; doi:10.3390/sports14050198)
Supplement: Supplementary file 1 [file sports-14-00198-s001.zip › Sup Materials 1 FPT Protocols.pdf]

Supplementary Materials 1: Table 1: FTP Protocols

Test	Equipment	Testing Process and Scoring	Indication for repeat test
**Anterior Reach (AR)**	Tape Measure<br>Tape to mark floor<br>1x camera<br>1x tripod<br>Y Balance Testing Kit	3 practice trials on each leg<br>Subject selects leg to test first<br>3 measured trials on one leg and then repeated on other leg<br>Distance pushed measured to nearest 0.5cm<br>Scoring completed at time of testing	Kicking push box<br>Not returning to starting position under control<br>Touching down during reach<br>Foot on top of stance plate
**Single Leg Drop Vertical Jump (DVJL)**	Tape Measure<br>Tape to mark floor<br>1 x camera<br>1 x tripod<br>35cm plyo box<br>Landing area marked 30cm in front of box with a strip of tape	Up to 3 practice reps each side with minimum of 1 minute rest<br>Subject selects leg to test first<br>Completes 3 measured trials on first leg and then repeats on other side<br>Landing to be held for at least 2 seconds on completion of DVJ<br>Scoring completed post testing using video footage and My Jump Lab App to calculate:<br>• Jump height<br>• Contact Time<br>• Flight Time<br>• Reactive strength index (RSI)	Loss of balance – steps out of landing<br>Extra hop on landing<br>Touching down with either contralateral leg or with hand

**Single Hop for Distance (SHFD)**	Tape Measure<br>Tape to mark floor<br>1 x camera<br>1 x tripod<br>Tape measure set to 250cm and secured to the floor with tape	Up to 3 practice reps each side with minimum of 1 minute rest<br>Subject selects leg to test first<br>Completes 3 measured trials on first leg and then repeats on other side<br>Landing to be held for at least 2 seconds<br>Measurement taken from heel of landing foot<br>Distance measured to the nearest cm<br>Scoring completed at time of testing	Loss of balance – steps out of landing<br>Extra hop on landing<br>Touching down with either contralateral leg or with hand
**Side Hop (SH)**	Tape Measure<br>Tape to mark floor<br>2 lines of 1metre 40cm apart<br>1 x camera<br>1 x tripod<br>Timer	Up to 10secs practice each side with minimum of 1 minute rest before completing testing<br>Completes 1 set on each leg<br>Self-selected rest between each side minimum of 1min<br>Subject selects leg to test first<br>Scoring completed post testing using video footage to calculate:<br>Total Hops,<br>Total Errors,<br>Adjusted Score (total hops – total errors)<br>% error (total errors/total hops x 100)	Loss of balance during the test<br>Forgets to keep hands on hips
