# Supplementary material for: Establishing the Reliability of a Functional Performance Test Battery That Incorporates the QASLS Tool in Pre-Elite Female Field Hockey Players
Source: Sports (Basel). 2026 May 12;14(5):198. doi: 10.3390/sports14050198 (PMC13210639; doi:10.3390/sports14050198)
Supplement: Supplementary file 1 [file sports-14-00198-s001.zip › Sup Materials 3 Stata Syntax.pdf]

**Supplementary Materials 2: Syntax for STATA 18 Statistical Analysis**

**Calculating ICC2,1 and ICC2,k in Stata 18**

**ICC2,1 Calculation**
To calculate ICC2,1 (single measurement, absolute agreement) in Stata 18, we used the following syntax:

**icc hop_distance participant time, absolute**

**Example Dataset**

```
    +-----------------------------+
    | participant   time   hop_distance   |
    |-----------------------------|
 1. |      1      1   150      1   |
 2. |      1      2   155      2   |
 3. |      1      3   160      3   |
 4. |      1      4   158      4   |
    +-----------------------------+
```

**ICC2,k Calculation**

To calculate ICC2,k (average measurement, absolute agreement) in Stata 18, we used the following syntax:

**icc hop_distance participant time, absolute k(3)**

**Example Dataset**

```
    +-----------------------------+
    | participant   time   hop_distance   |
    |-----------------------------|
 1. |      1      1   150      1   |
 2. |      1      2   155      2   |
 3. |      1      3   160      3   |
 4. |      1      4   158      4   |
    +-----------------------------+
```

**Calculating SEM, SDD in Stata 18**

***1. Input Data***

```
clear
input subject l1 l2 l3
1 10 12 11
2 15 14 16
3 20 19 21
4 25 26 24
5 30 28 29
6 35 34 36
7 40 39 41
8 45 46 44
9 50 48 49
10 55 54 56
end
```

***2. Calculate Differences and Means for Each Pair***

```
gen diff12 = l1 - l2
gen mean_measurement12 = (l1 + l2) / 2

gen diff13 = l1 - l3
gen mean_measurement13 = (l1 + l3) / 2

gen diff23 = l2 - l3
gen mean_measurement23 = (l2 + l3) / 2
```

***3. Calculate Mean and Standard Deviation of Differences for Each Pair***

```
summarize diff12, meanonly
local mean_diff12 = r(mean)
summarize diff12, detail
local sd_diff12 = r(sd)

summarize diff13, meanonly
local mean_diff13 = r(mean)
summarize diff13, detail
local sd_diff13 = r(sd)

summarize diff23, meanonly
local mean_diff23 = r(mean)
summarize diff23, detail
local sd_diff23 = r(sd)
```

***4. Calculate SEM and SDD for Each Pair***

```
local sem12 = `sd_diff12' / sqrt(2)
local sem13 = `sd_diff13' / sqrt(2)
local sem23 = `sd_diff23' / sqrt(2)

local sdd12 = 1.96 * sqrt(2) * `sd_diff12'
local sdd13 = 1.96 * sqrt(2) * `sd_diff13'
local sdd23 = 1.96 * sqrt(2) * `sd_diff23'
```

***5. Display Results in the Command Window***

```
display "Pair of Measurements         | SEM Value | SDD Value"
display "-----------------------------------------------------"
display "l1 and l2                  | " %9.3f `sem12' " | " %9.3f `sdd12'
display "l1 and l3                  | " %9.3f `sem13' " | " %9.3f `sdd13'
display "l2 and l3                  | " %9.3f `sem23' " | " %9.3f `sdd23'
```
