# Supplementary material for: Establishing the Reliability of a Functional Performance Test Battery That Incorporates the QASLS Tool in Pre-Elite Female Field Hockey Players
Source: Sports (Basel). 2026 May 12;14(5):198. doi: 10.3390/sports14050198 (PMC13210639; doi:10.3390/sports14050198)
Supplement: Supplementary file 1 [file sports-14-00198-s001.zip › Sup Materials 4.QASLS PEA docx.pdf]

Supplementary Materials 4:Table 4 Intra and Inter-Rater Reliability of Individual QASLS Component assessed by PEA%

	Anterior Reach				Drop Vertical Jump Landing				Side Hop				Single Hop for Distance			
**QASLS Component**	**R1**	**R1v2**	**R1v3**	**R2v3**	**R1**	**R1v2**	**R1v3**	**R2v3**	**R1**	**R1v2**	**R1v3**	**R2v3**	**R1**	**R1v2**	**R1v3**	**R2v3**
**Left 1**	100.0	100.0	100.0	100.0	100.0	96.7	100.0	96.7	100.0	98.3	98.3	96.7	90.0	76.7	73.3	70.0
**Right 1**	100.0	100.0	96.7	96.7	100.0	100.0	100.0	100.0	98.3	98.3	96.7	98.3	93.3	83.3	90.0	86.7
**Left 2**	93.3	90.0	83.3	80.0	100.0	90.0	70.0	80.0	96.7	93.3	83.3	85.0	100.0	93.3	90.0	83.3
**Right 2**	93.3	86.7	80.0	86.7	90.0	93.3	96.7	93.3	91.7	95.0	85.0	90.0	93.3	83.3	83.3	80.0
**Left 3**	100.0	90.0	83.3	76.7	96.7	90.0	86.7	86.7	80.0	88.3	85.0	66.7	90.0	73.3	86.7	73.3
**Right 3**	90.0	76.7	90.0	86.7	93.3	80.0	86.7	73.3	86.7	88.3	80.0	76.7	93.3	80.0	86.7	93.3
**Left 4**	90.0	73.3	76.7	63.3	76.7	86.7	93.3	80.0	83.3	78.3	75.0	63.3	83.3	66.7	80.0	80.0
**Right 4**	86.7	70.0	80.0	70.0	86.7	86.7	93.3	76.7	78.3	88.3	83.3	71.7	93.3	63.3	76.7	60.0
**Left 5**	93.3	80.0	80.0	86.7	100.0	90.0	93.3	96.7	93.3	96.7	93.3	96.7	100.0	96.7	90.0	93.3
**Right 5**	90.0	76.7	76.7	86.7	93.3	96.7	90.0	93.3	90.0	91.7	88.3	86.7	86.7	90.0	90.0	86.7
**Left 6**	96.7	96.7	93.3	90.0	90.0	90.0	93.3	83.3	66.7	86.7	81.7	76.7	90.0	90.0	93.3	83.3
**Right 6**	93.3	93.3	86.7	93.3	90.0	86.7	76.7	76.7	66.7	80.0	88.3	68.3	90.0	83.3	93.3	83.3
**Left 7**	93.3	83.3	90.0	80.0	96.7	86.7	90.0	96.7	90.0	88.3	80.0	85.0	93.3	96.7	93.3	96.7
**Right 7**	93.3	73.3	83.3	76.7	96.7	93.3	90.0	90.0	88.3	80.0	78.3	88.3	83.3	80.0	80.0	86.7
**Left 8**	96.7	93.3	93.3	100.0	96.7	96.7	96.7	100.0	91.7	93.3	95.0	91.7	96.7	96.7	96.7	100.0
**Right 8**	100.0	90.0	86.7	76.7	100.0	96.7	96.7	93.3	88.3	93.3	91.7	91.7	90.0	83.3	86.7	96.7
**Left 9**	100	100.0	90.0	83.3	100	100.0	100.0	100.0	100	100.0	98.3	98.3	100	100.0	86.7	86.7
**Right 9**	100	100.0	86.7	86.7	100	100.0	100.0	100.0	100	100.0	96.7	96.7	100	100.0	96.7	96.7
**Left 10**	100	100.0	100.0	100.0	100	100.0	100.0	100.0	100	100.0	100.0	100.0	100	100.0	100.0	100.0
**Right 10**	100	100.0	100.0	100.0	100.0	100.0	100.0	100.0	100	100.0	100.0	100.0	100	100.0	100.0	100

Substantial Agreement

QASLS Components Numbered as per scoring sheet

R1= Rater 1, R2 = Rater 2, R3= Rater 3
